# Supplementary material for: Diagnostic role of heart rate variability in breast cancer and its relationship with peripheral serum carcinoembryonic antigen
Source: PLoS One. 2023 Apr 6;18(4):e0282221. doi: 10.1371/journal.pone.0282221 (PMC10079040; doi:10.1371/journal.pone.0282221)
Supplement: S1 Table — In the grouping column, “1” represents the breast cancer group and “0” represents the control group. (PDF) [file pone.0282221.s002.pdf]

	Grouping[a]	Shapiro–Wilk	
		Freedom	P-value
Age(year)	0	18	0.682
	1	19	0.132
Weight(kg)	0	18	0.117
	1	19	0.457
Height(cm)	0	18	0.211
	1	19	0.105
BMI(kg/m^2)	0	18	0.202
	1	19	0.956
BMR(％)	0	18	0.186
	1	19	0.016
ALT(IU/L)	0	18	0.001
	1	19	0.000
AST(IU/L)	0	18	0.040
	1	19	0.000
AST/ALT	0	18	0.002
	1	19	0.001
Urea(mmol/L)	0	18	0.049
	1	19	0.018
TC(mmol/L)	0	18	0.673
	1	19	0.564
TBIL(μmol/L)	0	18	0.712
	1	19	0.041
TP(g/L)	0	18	0.167
	1	19	0.428
Ca(mmol/L)	0	18	0.682
	1	19	0.018
GLO(g/L)	0	18	0.383
	1	19	0.047
TG(mmol/L)	0	18	0.007
	1	19	0.018
ALB(g/L)	0	18	0.639
	1	19	0.054
ALB/GLO	0	18	0.369
	1	19	0.270
DBIL(μmol/L)	0	18	0.223
	1	19	0.990
ALP(IU/L)	0	18	0.560
	1	19	0.000
Crea(μmol/L)	0	18	0.278
	1	19	0.057
Glu(mmol/L)	0	18	0.003
	1	19	0.000

IBIL(μmol/L)	0	18	0.427
	1	19	0.048
CEA(ng/ml)	0	18	0.048
	1	19	0.003
Total TP(ms^2)	0	18	0.008
	1	19	0.169
Total VLF(ms^2)	0	18	0.058
	1	19	0.095
Total LF(ms^2)	0	18	0.002
	1	19	0.018
Total HF(ms^2)	0	18	0.000
	1	19	0.002
Total SDNN(ms)	0	18	0.972
	1	19	0.048
Total SDNNin(ms)	0	18	0.000
	1	19	0.134
Total rMSSD(ms)	0	18	0.001
	1	19	0.115
Total pNN50(%)	0	18	0.000
	1	19	0.003
Awake TP(ms^2)	0	18	0.002
	1	19	0.039
Awake VLF(ms^2)	0	18	0.006
	1	19	0.027
Awake LF(ms^2)	0	18	0.000
	1	19	0.002
Awake HF(ms^2)	0	18	0.000
	1	19	0.000
Awake SDNN(ms)	0	18	0.156
	1	19	0.452
Awake SDNNin(ms)	0	18	0.007
	1	19	0.174
Awake rMSSD(ms)	0	18	0.001
	1	19	0.046
Awake pNN50(%)	0	18	0.000
	1	19	0.000
Sleep TP(ms^2)	0	18	0.025
	1	19	0.145
Sleep VLF(ms^2)	0	18	0.118
	1	19	0.070
Sleep LF(ms^2)	0	18	0.000
	1	19	0.014
Sleep HF(ms^2)	0	18	0.000
	1	19	0.007

Sleep SDNN(ms)	0	18	0.820
	1	19	0.031
Sleep SDNNin(ms)	0	18	0.190
	1	19	0.014
Sleep rMSSD(ms)	0	18	0.006
	1	19	0.051
Sleep pNN50(%)	0	18	0.001
	1	19	0.002
