# Supplementary material for: Diagnostic role of heart rate variability in breast cancer and its relationship with peripheral serum carcinoembryonic antigen
Source: PLoS One. 2023 Apr 6;18(4):e0282221. doi: 10.1371/journal.pone.0282221 (PMC10079040; doi:10.1371/journal.pone.0282221)
Supplement: S2 Table — a All groups of this index followed a normal distribution and were expressed as x¯±s. Independent samples t-test was used for comparison between groups. b At least one of the groups of this indicator did not follow a normal distribution, denoted by M (P25, P75), and comparisons between groups were made using rank sum test. (PDF) [file pone.0282221.s003.pdf]

Variables	Control group	Breast cancer group	F /Z-value	P-value
CEA[b](ng/ml)	1.25(0.70,1.63)	2.70(1.60,5.20)	-3.984	<0.001
TP[b](ms^2)	1314.40(991.25,2072.00)	943.00(507.00,1686.00)	-1.565	0.118
Total VLF[a](ms^2)	963.91±387.15	767.97±429.63	1.033	0.155
Total LF[b](ms^2)	283.90(195.50,554.50)	213.40(80.00,421.70)	-2.006	0.045
Total HF[b](ms^2)	131.55(110.75,256.25)	104.50(56.00,222.90)	-1.337	0.181
Total SDNN[b](ms)	129.00(109.75,153.75)	129.00(79.00,158.00)	-0.502	0.616
Total SDNNin[b](ms)	49.00(39.75,63.25)	46.00(30.00,56.00)	-1.157	0.247
Total rMMSD[b](ms)	23.50(20.75,30.50)	23.00(18.00,33.00)	-0.624	0.533
Total pNN50[b](%)	4.50(2.00,10.25)	4.00(1.00,12.00)	-0.458	0.647
Awake TP[b](ms^2)	1298.50(1044.00,2033.75)	816.00(454.00,1519.80)	-2.127	0.033
Awake VLF[b](ms^2)	854.50(698.85,1124.00)	623.60(294.00,999.00)	-1.914	0.056
Awake LF[b](ms^2)	298.50(214.75,612.00)	152.00(84.00,344.00)	-2.340	0.019
Awake HF[b](ms^2)	91.50(83.75,174.00)	76.20(49.00,134.90)	-1.033	0.301
Awake SDNN[a](ms)	101.72±20.77	91.00±31.08	3.511	0.228
Awake SDNNin[b](ms)	42.50(39.75,53.25)	41.00(31.00,55.00)	-0.715	0.475
Awake rMSSD[b](ms)	21.00(17.75,29.25)	20.00(17.00,28.00)	-0.685	0.493
Awake pNN50[b](%)	2.50(1.00,8.25)	2.00(1.00,6.00)	-0.460	0.645
Sleep TP[b](ms^2)	1455.10(1003.75,2328.00)	1256.00(616.00,2098.00)	-0.851	0.395
Sleep VLF[a](ms^2)	979.80±446.43	827.61±461.47	0.705	0.315
Sleep LF[b](ms^2)	317.55(156.25,516.50)	260.00(88.00,489.40)	-1.215	0.224

Sleep HF[b](ms^2)	187.75(100.75,422.50)	124.80(53.00,331.40)	-1.094	0.274
Sleep SDNN[b](ms)	106.50(83.50,140.25)	125.00(64.00,162.00)	-0.304	0.761
Sleep SDNNin[b](ms)	50.50(39.00,63.25)	55.00(34.00,70.00)	-0.061	0.952
Sleep rMSSD[b](ms)	28.50(22.50,39.00)	28.00(18.00,46.00)	-0.395	0.693
Sleep pNN50[b](％)	7.00(2.75,19.75)	6.00(1.00,21.00)	-0.564	0.573
