# Supplementary material for: Diagnostic role of heart rate variability in breast cancer and its relationship with peripheral serum carcinoembryonic antigen
Source: PLoS One. 2023 Apr 6;18(4):e0282221. doi: 10.1371/journal.pone.0282221 (PMC10079040; doi:10.1371/journal.pone.0282221)
Supplement: S3 Table — (PDF) [file pone.0282221.s004.pdf]

Step	Chi-square value	Freedom	P-value
First	9.309	7	0.231
