# Supplementary material for: Diagnostic role of heart rate variability in breast cancer and its relationship with peripheral serum carcinoembryonic antigen
Source: PLoS One. 2023 Apr 6;18(4):e0282221. doi: 10.1371/journal.pone.0282221 (PMC10079040; doi:10.1371/journal.pone.0282221)
Supplement: S4 Table — SE, standard error; D-W value, the indicators of Durbin-Watson test. -7.550E-5 represents -7.550×10^−5^. (PDF) [file pone.0282221.s005.pdf]

Model	Sum of squares	Freedom	Mean square	F-value	P-value
Regression	3.373	4	0.843	4.597	0.005
Residual	5.870	32	0.183	-	-
Total	9.243	36	-	-	-

R-value	R^2	Adjusted R^2	SE	D-W value
0.604	0.365	0.286	0.428	0.809

Model	Non standardized coefficient		Standardized coefficient	t	P-value	Collinearity statistics	
	B-value	SE				Tolerance	VLF
CEA(ng/ml)	0.098	0.030	0.482	3.241	0.003	0.897	1.115
Total LF(ms$^{2)}$	0.002	0.001	0.913	1.587	0.122	0.060	16.677
Awake TP(ms^2)	-7.550E-5	0.000	-0.124	-0.265	0.793	0.090	11.072
Awake LF(ms^2)	-0.002	0.001	-0.971	-1.559	0.129	0.051	19.528
