# Supplementary material for: Diagnostic role of heart rate variability in breast cancer and its relationship with peripheral serum carcinoembryonic antigen
Source: PLoS One. 2023 Apr 6;18(4):e0282221. doi: 10.1371/journal.pone.0282221 (PMC10079040; doi:10.1371/journal.pone.0282221)
Supplement: S5 Table — (PDF) [file pone.0282221.s006.pdf]

Variables	r-value	P-value
Total LF(ms^2)	-0.387	0.018
Awake TP(ms^2)	-0.425	0.009
Awake LF(ms^2)	-0.426	0.009
