# Supplementary material for: Diagnostic role of heart rate variability in breast cancer and its relationship with peripheral serum carcinoembryonic antigen
Source: PLoS One. 2023 Apr 6;18(4):e0282221. doi: 10.1371/journal.pone.0282221 (PMC10079040; doi:10.1371/journal.pone.0282221)
Supplement: S6 Table — (PDF) [file pone.0282221.s007.pdf]

Model	B-value	SE	Wald	P-value	Exp(B)
Awake TP(ms$^{2)}$	0.002	0.002	0.902	0.342	1.002(0.998-1.006)
Awake LF(ms$^{2)}$	-0.008	0.007	1.295	0.255	0.992(0.979-1.006)
CEA(ng/ml)	1.324	0.595	4.954	0.026	3.760(1.171-12.069)
